# Predictive value of six anthropometric indicators for prevalence and mortality of obstructive sleep apnoea asthma and COPD using NHANES data

**DOI:** 10.1038/s41598-025-99490-y

**Published:** 2025-05-09

**Authors:** Jingdi Hu, Songwen Tang, Qijiang Zhu, Huai Liao

**Affiliations:** 1 Department of Pulmonary and Critical Care Medicine, The First Affiliated Hospital of Sun Yat-Sen University, Guangzhou, Guangdong China; 2 Department of Endocrinology and Diabetes Center, The First Affiliated Hospital of Sun Yat-Sen University, Guangzhou, Guangdong China

**Keywords:** The US National Health and Nutrition Examination Survey, Obesity, Obstructive sleep apnoea, Asthma, Chronic obstructive pulmonary disease, Mortality

## Abstract

Obesity is linked to a greater risk of respiratory diseases. Due to limitations in body mass index (BMI), alternative anthropometric indicators have been developed to reflect body fat distribution. This study compares six anthropometric measures—BMI, waist circumference (WC), the waist-to-height ratio (WHtR), the body roundness index (BRI), the body shape index (ABSI), and the weight-adjusted waist index (WWI)—and their relationships with the prevalence and mortality of obstructive sleep apnoea (OSA), asthma, and chronic obstructive pulmonary disease (COPD) in the US population. Data from four NHANES cycles were analyzed. Multivariable logistic regression assessed the cross-sectional associations between the six anthropometric measures and disease prevalence. Mortality associations were analysed via Cox proportional hazards models, and time‒dependent ROC curve was utilised to evaluate the predictive performance of the significant marker for mortality. BMI, WC, WWI, BRI, ABSI, and WHtR were positively correlated with the prevalence of OSA, and COPD. For asthma, BMI, WC, BRI, and WHtR were positively associated with prevalence, while ABSI and WWI were negatively associated. Concerning mortality, higher WC and BMI were associated with better survival in the OSA and COPD groups, whereas elevated WWI and ABSI were linked to greater mortality risk in the participants with OSA symptoms. An increase of one standard deviation (SD) in the ABSI resulted in an 18% increase in mortality (95% CI 1.09–1.27) for the OSA population. The area under the curve (AUC) for ABSI was 0.752 for 3-year, 0.755 for 5-year, and 0.744 for 10-year mortality. Novel anthropometric indicators, including WWI, BRI, ABSI, and WHtR, show positive associations with the prevalence of OSA, and COPD, alongside traditional measures like BMI and WC. However, WWI and ABSI were more limited in their association with asthma prevalence. Longitudinal analyses revealed that traditional anthropometric indicators such as BMI and WC were negatively associated with mortality risks in the OSA and COPD, supporting the "obesity paradox." ABSI, however, emerged as a significant mortality predictor for OSA, providing a more nuanced view of central obesity’s impact on mortality. However, in COPD patients, routine anthropometric measurements may not fully capture the effects of obesity.

## Introduction

Obesity has become a global health crisis, characterized by a dramatic rise in prevalence and its systemic impact on chronic diseases (e.g., diabetes, cardiovascular conditions) and respiratory illnesses. In 2022, obesity affected approximately 16% of the global adult population. Between 1990 and 2022, the worldwide prevalence of obesity increased by more than twofold^1^. Increased risk of several chronic diseases is associated with the prevalence of obesity^2^. Furthermore, obesity has been linked to airway inflammation, reduced lung function, and the occurrence and worsening of respiratory diseases, including obstructive sleep apnoea (OSA)^3^, asthma^4^, and chronic obstructive pulmonary disease (COPD)^5^. These effects can be attributed to the fact that obesity leads to metabolic dysregulation and chronic inflammation, which influence overall body health, as well as fat accumulation in the abdominal cavity, which negatively impacts lung function^6,7^.

Body mass index (BMI) remains the most widely used measure to assess obesity, but its limitations have spurred interest in alternative metrics that better capture health risks. The World Health Organization (WHO) classifies adults with a BMI of 30 or higher as obese^1^. However, BMI has been shown to be poor at distinguishing between fat mass and lean mass. Recent studies have increasingly focused on body fat distribution rather than merely excess weight^8^. Dual-energy X-ray absorptiometry (DXA) provides highly accurate body composition assessments, but its complexity and high cost hinder widespread use, but its complexity and high cost limit widespread use^9^. Waist circumference (WC) serves as a simpler, more accessible proxy for abdominal fat. It has been advocated for inclusion as a routine clinical measurement^10^. However, WC is highly correlated with BMI, which may also give rise to the obesity paradox, wherein obesity appears to have protective effects in some conditions^11^. To address this issue, the body shape index (ABSI) and waist-to-height ratio (WHtR) have been developed to capture the added value of WC; these indices correspond to visceral fat and can predict mortality^12,13^. The body roundness index (BRI), assuming the body is elliptical and calculates its eccentricity, serves as another promising measure of abdominal adiposity. It has been studied for its value in predicting cardiovascular events^14^, mortality^15^, and the deterioration of lung function^16,17^. The weight-adjusted waist index (WWI) also undergoes evaluation alongside WHtR, BMI, and WC, demonstrating the strongest association with sarcopenic obesity^18^.

Several surveys have examined and compared the role of various anthropometric measures of lung function, which are related to the onset and prognosis of respiratory diseases. For example, a longitudinal study in Taiwan found that elevated levels of BRI, WHtR, and BMI were linked to a decline in lung function^16^. However, the impact of various anthropometric measures on the prevalence and mortality of respiratory diseases remains underexplored.

We hypothesized that these novel indicators—WWI, BRI, ABSI, and WHtR—would outperform traditional anthropometric measures (BMI and WC) in predicting the prevalence or mortality of respiratory disease. To test this, the study sought to elucidate the impacts of diverse anthropometric measures on the prevalence and mortality of diseases such as OSA, asthma, and COPD. Furthermore, it aimed to identify the most accurate metric for assessing and addressing adiposity in individuals with these chronic respiratory diseases.

## Methods

### Study population

**Fig. 1:**
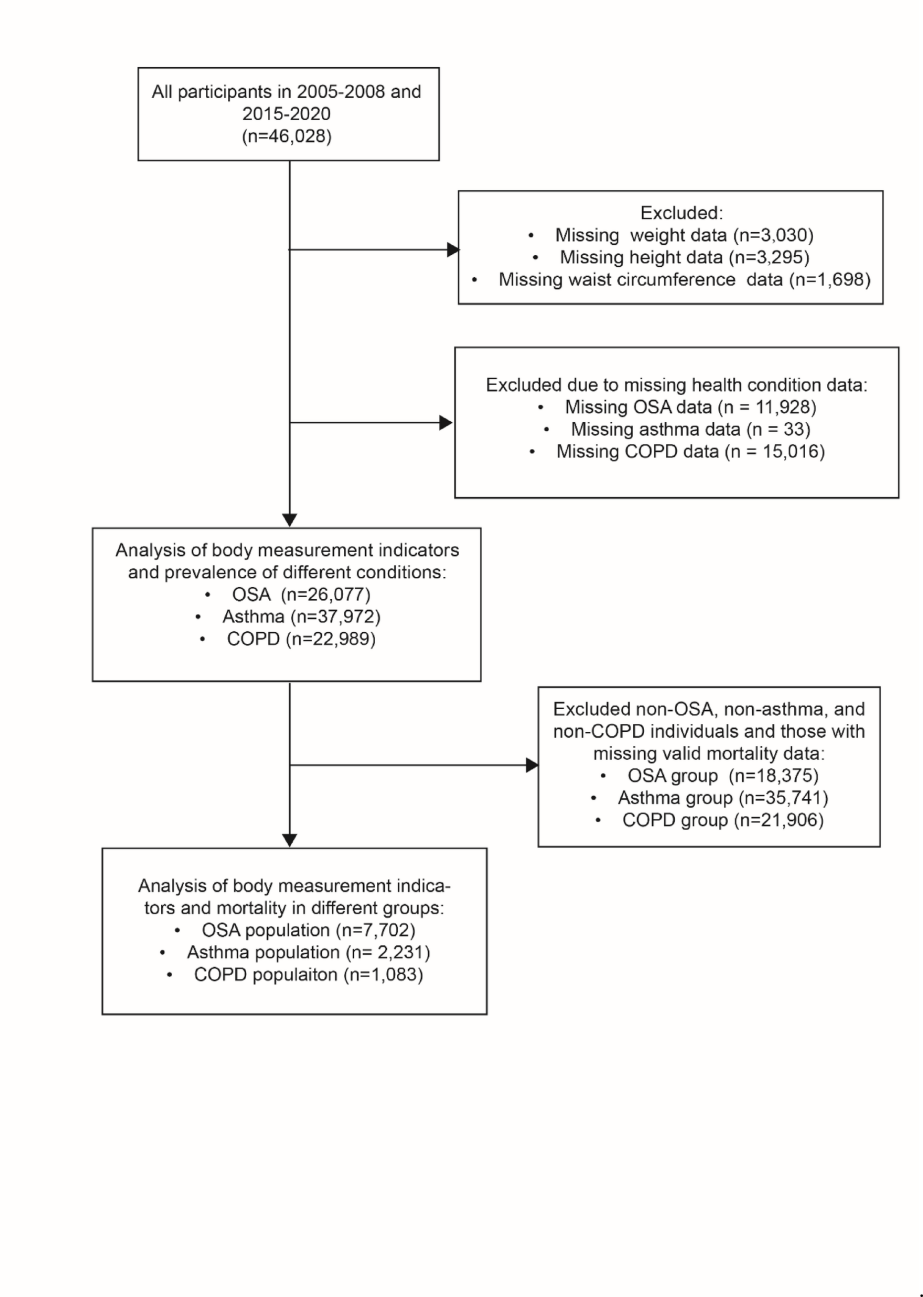
Flow chart of the selection procedure.

Four cycles of data (2005–2006, 2007‒2008, 2015‒2016, and 2017‒2020) came from the US National Health and Nutrition Examination Survey (NHANES) database. Initially, the four-cycle NHANES data included 46,028 participants. Individuals with missing data on key anthropometric measures—such as weight (n = 3030), height (n = 3295), and waist circumference (n = 1698)—were excluded. The remaining participants were grouped based on available data for OSA, asthma, and COPD, resulting in 26,077 with OSA-related data, 37,972 with asthma-related data, and 22,989 with COPD-related data. For the longitudinal analysis, additional exclusions were made to remove non-OSA, non-asthma, and non-COPD individuals, as well as those with missing valid mortality data. Ultimately, the analysis focused on 7702 participants with OSA, 2231 with asthma, and 1083 with COPD, all having complete body measurement and mortality data. Figure 1 presents a flow chart detailing the selection procedure of participants for this research.

### Assessment of OSA, asthma, and COPD

OSA symptoms were defined on the basis of individuals reporting snoring, gasping for air, experiencing pauses in breathing three or more times a week, or experiencing excessive daytime sleepiness on at least 16 days per month^19^. People were diagnosed with asthma if they had ever been told by doctors that they had asthma. People were diagnosed with COPD if they had ever been told by doctors that they had COPD, chronic bronchitis, or emphysema.

### Measurement of six body measurement indicators

Trained health professionals collected participants’ height, weight, and waist circumference (WC) data. From these measurements, six body measurement indicators—BMI, WWI, BRI, ABSI, and WHtR—were calculated via the following equations:

$$\begin{aligned} BMI & = weight/height^{2} \\ WWI & = WC/\sqrt {weight} \\ BRI & = 364.2 - 365.5 \times \sqrt {1 - \left( {WC/2\pi } \right)^{2} /\left( {0.5\;height} \right)^{2} } \\ ABSI & = WC/\left( {BMI^{2/3} \;height^{1/3} } \right) \\ WHtR & = WC/height \\ \end{aligned}$$

### Mortality

Mortality data were obtained from the National Death Index (NDI), which is linked with NHANES records by the National Center for Health Statistics. The analysis focused on all-cause mortality and monitoring starting from the participant’s enrolment date and continuing until death or December 31, 2019.

### Covariables

Based on the previous studies on obesity and chronic respiratory disease, this analysis included the following covariates that may influence the association between obesity and respiratory outcomes and mortality^20,21^. Demographic variables such as age, gender, race, education, and poverty-income ratio were gathered through interviews. The estimated glomerular filtration rate (eGFR) was calculated from the re-calibrated serum creatinine using the CKD-EPI equation, with chronic kidney disease (CKD) defined as an eGFR below 60 mL/min/1.73 m^2^. Diabetes was identified based on any of the following: self-reported physician diagnosis, use of diabetes medication or insulin, fasting plasma glucose ≥ 7.0 mmol/L, or HbA1c ≥ 6.5%. Hypertension was defined by either a self-reported diagnosis, use of antihypertensive medication, or an average systolic blood pressure of 140 mmHg or above and/or an average diastolic blood pressure of 90 mmHg or above. Cardiovascular disease (CVD) was defined by a history of heart attack, angina, coronary heart disease, or stroke. Smoking status classified individuals as current smokers, quitters, and never smokers. Binge drinking is defined as alcohol consumption ≥ 4 drinks on one occasion for females, or ≥ 5 drinks on one occasion for males. Alcohol consumption was categorized as 4 levels: binge drinking daily; heavy drinking (defined as consuming ≥ 3 drinks per day for females, or ≥ 4 drinks per day for males, or binge drinking on five or more days per month); moderate drinking (defined as consuming ≥ 2 drinks per day for females, or ≥ 3 drinks per day for males, or binge drinking on two or more days per month); mild drinking (not meeting the above criteria); or never drinking.

### Statistical analysis

Missing covariable values were imputed via the random forest algorithm implemented via missRanger in R (version 4.3.3). This method uses chained random forest models to iteratively impute missing values, leveraging the observed data from other variables to predict missing values. This study took into account the complex sampling design and sampling weights of the NHANES database by employing a full sample exam weight. Continuous variable outcomes are reported as survey-weighted means (standard errors), with differences between groups calculated using survey-weighted linear regression. Categorical variables are displayed as unweighted counts alongside their corresponding weighted percentages, and group differences were assessed using the survey-weighted Chi-square test.

The anthropometric indicators were assessed both as continuous variables, with each standard deviation increment represented, and as categorical variables, with the quartiles delineated. The tests for linear and p-values for trend were calculated to assess whether there was a significant linear association between the quartiles of the anthropometric indicators and the outcomes, with the median values of each quartile assigned to the variables. Multivariable logistic regression models investigated the associations of six anthropometric indicators with the prevalence of OSA, asthma, and COPD. The findings are reported as odds ratios (ORs) and their 95% confidence intervals (CIs). Multivariable Cox proportional hazards models assessed the relationships between the examined factors and mortality in the overall population, as well as in the OSA, asthma, and COPD subgroups. The results are shown as the hazard ratios (HRs) alongside 95% CIs. A time-dependent ROC curve evaluated the predictive performance of ABSI for mortality at different time points among the OSA population. Furthermore, Kaplan–Meier survival curves for all-cause mortality were generated, and the differences were assessed using the log-rank test. A stratified analysis further investigated the relationship between ABSI and mortality, considering factors such as sex, age, race, PIR, diabetes, hypertension, CVD, CKD, smoking status, and alcohol consumption.

All data analyses were conducted via R programming (version 4.3.3) and EmpowerStats software (X&Y Solutions, Inc.), with statistical significance set at a *p*-value below 0.05.

## Results

### Characteristics of the population included in the cross-sectional analysis

Table 1 outlines the basic characteristics of the participants, comparing those with OSA, asthma, and COPD to healthy individuals. OSA participants were older (47.47 years) and predominantly male (54.81%) compared to healthy participants (43.10 years, 42.76% male). Asthma participants were younger (36.06 years) with a slightly higher proportion of females (54.14%) compared to healthy individuals (38.77 years, 50.42% female). COPD participants were older (55.38 years) and more likely to be female (60.89%) compared to healthy participants (46.52 years, 51.01% female).

**Table 1:** Weighted baseline characteristics of the participants in the cross-sectional study.

	OSA			Asthma			COPD		
	No (n = 13,870)	Yes (n = 12,207)	*p*-value	No (n = 32,187)	Yes (n = 5785)	*p*-value	No (n = 21,187)	Yes (n = 1802)	*p*-value
Demographic data									
Age, year	43.10 (0.32)	47.47 (0.31)	** < 0.0001**	38.77 (0.33)	36.06 (0.41)	** < 0.0001**	46.52 (0.29)	55.38 (0.55)	** < 0.0001**
Gender			** < 0.0001**			**0.0036**			** < 0.0001**
Male	6168 (42.76%)	6578 (54.81%)		15,955 (49.58%)	2815 (45.86%)		10,337 (48.99%)	817 (39.11%)	
Female	7702 (57.24%)	5629 (45.19%)		16,232 (50.42%)	2970 (54.14%)		10,850 (51.01%)	985 (60.89%)	
Race			0.4047			** < 0.0001**			** < 0.0001**
White	5371 (66.32%)	4732 (66.45%)		11,474 (63.47%)	2089 (63.93%)		8179 (66.23%)	1033 (76.55%)	
Black	3347 (11.50%)	2858 (11.37%)		7641 (11.52%)	1736 (14.35%)		4952 (11.37%)	368 (9.23%)	
Hispanic	3546 (14.20%)	3359 (14.75%)		9429 (16.63%)	1383 (13.80%)		5662 (14.63%)	267 (7.05%)	
Others	1606 (7.98%)	1258 (7.43%)		3643 (8.38%)	577 (7.92%)		2394 (7.77%)	134 (7.17%)	
Education level			** < 0.0001**			**0.0001**			** < 0.0001**
Under high school	1149 (4.64%)	1279 (5.69%)		2822 (4.90%)	311 (3.24%)		2235 (5.44%)	191 (6.31%)	
High school	4945 (31.29%)	4688 (36.23%)		12,150 (34.19%)	2197 (32.61%)		7774 (33.58%)	786 (43.42%)	
Above high school	7776 (64.07%)	6240 (58.08%)		17,215 (60.90%)	3277 (64.15%)		11,178 (60.97%)	825 (50.27%)	
PIR	3.02 (0.04)	3.03 (0.04)	0.6814	2.96 (0.04)	2.81 (0.05)	**0.0004**	3.10 (0.04)	2.57 (0.06)	** < 0.0001**
Smoking status			** < 0.0001**			**0.0099**			** < 0.0001**
Never	9199 (63.14%)	6622 (50.98%)		23,552 (66.01%)	4126 (63.51%)		12,320 (56.62%)	511 (29.19%)	
Former	2549 (20.92%)	2985 (25.98%)		4692 (18.94%)	836 (19.17%)		4861 (24.18%)	656 (34.83%)	
Now	2122 (15.95%)	2600 (23.04%)		3943 (15.05%)	823 (17.32%)		4006 (19.20%)	635 (35.98%)	
Alcohol consumption			** < 0.0001**			0.4940			** < 0.0001**
Never	9591 (62.60%)	7318 (53.87%)		24,403 (65.85%)	4380 (65.59%)		13,041 (56.45%)	1031 (55.00%)	
Mild	2289 (21.54%)	2226 (21.77%)		3856 (17.96%)	683 (17.55%)		4083 (23.10%)	229 (14.35%)	
Moderate	511 (4.65%)	497 (5.02%)		860 (4.09%)	148 (4.17%)		924 (5.23%)	51 (3.26%)	
Heavy	223 (1.95%)	242 (2.46%)		406 (1.94%)	59 (1.54%)		426 (2.38%)	29 (1.74%)	
Binge drinking daily	1256 (9.26%)	1924 (16.89%)		2662 (10.16%)	515 (11.15%)		2713 (12.84%)	462 (25.65%)	
Body measurement data									
Weight, kg	76.81 (0.35)	87.69 (0.34)	** < 0.0001**	73.29 (0.30)	76.50 (0.56)	** < 0.0001**	82.52 (0.32)	84.69 (0.65)	**0.0009**
Height, cm	167.81 (0.12)	169.33 (0.15)	** < 0.0001**	161.91 (0.17)	163.00 (0.30)	**0.0016**	168.63 (0.12)	166.56 (0.31)	** < 0.0001**
BMI, kg/m^2^	27.20 (0.12)	30.51 (0.10)	** < 0.0001**	26.86 (0.09)	28.02 (0.18)	** < 0.0001**	28.92 (0.10)	30.47 (0.22)	** < 0.0001**
WC, cm	93.86 (0.32)	103.04 (0.28)	** < 0.0001**	91.89 (0.27)	94.15 (0.46)	** < 0.0001**	98.80 (0.28)	103.74 (0.54)	** < 0.0001**
WWI	10.76 (0.02)	11.06 (0.01)	** < 0.0001**	10.98 (0.01)	10.95 (0.02)	**0.0919**	10.93 (0.01)	11.35 (0.03)	** < 0.0001**
BRI	5.23 (0.04)	6.33 (0.04)	** < 0.0001**	5.35 (0.03)	5.65 (0.06)	** < 0.0001**	5.81 (0.04)	6.70 (0.08)	** < 0.0001**
ABSI	0.81 (0.00)	0.82 (0.00)	** < 0.0001**	0.81 (0.00)	0.80 (0.00)	**0.0004**	0.81 (0.00)	0.83 (0.00)	** < 0.0001**
WHtR	0.56 (0.00)	0.61 (0.00)	** < 0.0001**	0.57 (0.00)	0.58 (0.00)	** < 0.0001**	0.59 (0.00)	0.62 (0.00)	** < 0.0001**
Comorbidity									
Hypertension	4702 (31.28%)	5962 (45.38%)	** < 0.0001**	9072 (30.88%)	1664 (31.84%)	0.3477	9360 (39.02%)	1129 (56.59%)	** < 0.0001**
Diabetes	1783 (10.03%)	2618 (16.73%)	** < 0.0001**	3740 (10.93%)	746 (11.84%)	0.1863	3790 (13.19%)	553 (24.44%)	** < 0.0001**
CVD	981 (5.89%)	1296 (8.76%)	** < 0.0001**	1825 (5.71%)	449 (7.40%)	**0.0005**	1790 (6.57%)	486 (22.65%)	** < 0.0001**
CKD	993 (6.05%)	899 (5.93%)	0.7613	1652 (5.04%)	241 (3.86%)	**0.0025**	1639 (6.06%)	255 (11.05%)	** < 0.0001**

Continuous variables are presented as survey-weighted means (SEs), and *p-*value was calculated by survey-weighted linear regression. Categorical variables are presented as unweighted counts (weighted percentages), and *p-*value was calculated by survey-weighted Chi-square test.* P* < 0.005 was showed in bold.

PIR, poverty-income ratio; BMI, body mass index; WC, waist circumference; WWI, weight-adjusted-waist index; BRI, body roundness index; ABSI, body shape index; WHtR, waist‒height ratio; CVD, cardiovascular disease; CKD, chronic kidney disease; OSA, obstructive sleep apnoea; COPD, chronic obstructive pulmonary disease.

OSA, asthma, and COPD participants exhibited lower education levels and PIR compared to their healthy counterparts. Smoking and alcohol consumption were more prevalent in all three disease groups. Regarding anthropometric measurements, BMI, WC, BRI, and WHtR were elevated in all disease groups, while ABSI was lower in asthma participants, and WWI showed no significant difference between asthma and healthy individuals. Comorbidities such as hypertension, diabetes, CVD, and CKD were more common in participants with OSA and COPD compared to healthy individuals, with the highest prevalence observed in the COPD group.

### Associations between anthropometric indicators and OSA, asthma, and COPD

Table 2 illustrates the association between the six body measurement indicators and the prevalence of OSA, asthma, and COPD, adjusted for potential confounding variables, including sex, age, race, education level, PIR, diabetes, hypertension, CVD, CKD, smoking status, and alcohol consumption. All six indicators were significantly associated with an increased prevalence of OSA and COPD, with stronger associations observed for OSA. For each standard deviation (SD) increase, the prevalence of OSA rose by 64% for BMI (95% CI for OR: 1.60–1.69), 64% for WC (95% CI for OR: 1.59–1.68), 42% for WWI (95% CI for OR: 1.37–1.46), 62% for BRI (95% CI for OR: 1.58–1.67), 3% for ABSI (95% CI for OR: 1.00–1.06), and 65% for WHtR (95% CI for OR: 1.60–1.70). Among these, ABSI exhibited the weakest predictive value for OSA. For asthma, BMI, WC, BRI, and WHtR were positively associated with prevalence, while ABSI and WWI were negatively associated. Each SD increase in ABSI and WWI was linked to a 7% decrease in asthma prevalence (95% CI for OR: 0.90–0.96).

**Table 2:** Associations of six anthropometric indicators with OSA, asthma and COPD.

	Adjusted odds ratio (95% CI)^a^					
	Quartile 1	Quartile 2	Quartile 3	Quartile 4	*P* for trend	Per SD increment
OSA						
BMI	Ref	1.51 (1.40, 1.62)	2.25 (2.09, 2.43)	3.50 (3.24, 3.79)	< 0.0001	1.64 (1.60, 1.69)
WC	Ref	1.68 (1.55, 1.81)	2.50 (2.31, 2.70)	3.60 (3.32, 3.90)	< 0.0001	1.64 (1.59, 1.68)
WWI	Ref	1.73 (1.60, 1.87)	2.02 (1.86, 2.19)	2.44 (2.23, 2.67)	< 0.0001	1.42 (1.37, 1.46)
BRI	Ref	1.74 (1.61, 1.88)	2.47 (2.28, 2.67)	3.66 (3.37, 3.98)	< 0.0001	1.62 (1.58, 1.67)
ABSI	Ref	1.32 (1.23, 1.42)	1.29 (1.19, 1.39)	1.17 (1.07, 1.27)	0.0013	1.03 (1.00, 1.06)
WHtR	Ref	1.74 (1.61, 1.88)	2.46 (2.27, 2.66)	3.66 (3.37, 3.98)	< 0.0001	1.65 (1.60, 1.70)
Asthma						
BMI	Ref	1.45 (1.33, 1.59)	1.47 (1.34, 1.62)	1.94 (1.77, 2.14)	< 0.0001	1.27 (1.23, 1.31)
WC	Ref	1.56 (1.43, 1.71)	1.52 (1.37, 1.68)	1.91 (1.72, 2.12)	< 0.0001	1.31 (1.26, 1.36)
WWI	Ref	0.86 (0.79, 0.93)	0.87 (0.80, 0.95)	0.86 (0.79, 0.94)	0.0017	0.93 (0.90, 0.96)
BRI	Ref	0.92 (0.85, 1.00)	1.02 (0.93, 1.11)	1.40 (1.28, 1.53)	< 0.0001	1.21 (1.17, 1.25)
ABSI	Ref	0.83 (0.77, 0.90)	0.83 (0.76, 0.90)	0.80 (0.73, 0.88)	< 0.0001	0.93 (0.90, 0.96)
WHtR	Ref	0.92 (0.85, 1.00)	1.02 (0.93, 1.11)	1.40 (1.27, 1.53)	< 0.0001	1.20 (1.16, 1.24)
COPD						
BMI	Ref	0.85 (0.73, 0.99)	0.96 (0.82, 1.11)	1.44 (1.25, 1.67)	< 0.0001	1.21 (1.15, 1.27)
WC	Ref	0.87 (0.74, 1.02)	1.08 (0.93, 1.27)	1.48 (1.27, 1.72)	< 0.0001	1.23 (1.16, 1.29)
WWI	Ref	1.38 (1.15, 1.65)	1.35 (1.13, 1.62)	1.71 (1.42, 2.05)	< 0.0001	1.22 (1.15, 1.30)
BRI	Ref	0.94 (0.80, 1.11)	1.02 (0.87, 1.20)	1.52 (1.30, 1.77)	< 0.0001	1.24 (1.18, 1.30)
ABSI	Ref	0.88 (0.74, 1.05)	1.04 (0.88, 1.22)	1.22 (1.03, 1.44)	0.0013	1.12 (1.05, 1.18)
WHtR	Ref	0.94 (0.80, 1.11)	1.02 (0.87, 1.20)	1.51 (1.30, 1.77)	< 0.0001	1.24 (1.17, 1.30)

*OSA* obstructive sleep apnoea, *COPD* chronic obstructive pulmonary disease, *BMI* body mass index, *WC* waist circumference, *WWI* weight-adjusted-waist index, *BRI* body roundness index, *ABSI* body shape index, *WHtR* waist-to-height ratio.

^a^Adjusted for sex, age, race, education level, family poverty-income ratio, diabetes, hypertension, cardiovascular disease, chronic kidney disease, smoking status, and alcohol consumption.

### Characteristics of participants with eligible mortality data

Table 3 shows the population characteristics in the mortality analysis. Among the 7,702 OSA participants, 920 deaths were recorded during a median follow-up of 84.45 months. In the asthma cohort, 267 deaths occurred among 2,231 participants, with a median follow-up of 71.62 months. Similarly, in the COPD cohort, 314 deaths were documented among 1,083 participants, with a median follow-up of 71.82 months. Deceased individuals across all cohorts exhibited significant differences (*p* < *0.05*) in age, gender, race, and socioeconomic status compared to survivors. The deceased group tended to be older (OSA: 64.93 vs. 45.79 years; asthma: 63.22 vs. 41.46 years; COPD: 67.46 vs. 50.26 years), male (OSA: 58.6% vs. 55.0%; COPD: 53.1% vs. 32.5%), white (OSA: 78.1% vs. 66.9%; asthma: 76.5% vs. 70.5%; COPD: 83.0% vs. 75.9%), and have a lower PIR (OSA: 2.14 vs. 3.10; asthma: 2.21 vs. 2.97; COPD: 2.24 vs. 2.71). Additionally, tobacco and alcohol use were strongly associated with mortality: current and former smokers had higher mortality rates. Heavy and binge drinking correlated with mortality risk. Deceased individuals had elevated WC in OSA (104.00 vs. 102.57 cm; *p* = *0.043*) and asthma (103.15 vs. 99.62 cm; *p* = *0.009*). WWI, ABSI, and WHtR were significantly higher in deceased groups across all cohorts. Furthermore, individuals who died were more frequently affected by comorbidities such as hypertension, diabetes, CVD, and CKD.

**Table 3:** Weighted baseline characteristics of participants with eligible mortality data.

	OSA			Asthma			COPD		
	Alive (n = 6782)	Deceased (n = 920)	*p-*value	Alive (n = 1964)	Deceased (n = 267)	*p-*value	Alive (n = 769)	Deceased (n = 314)	*p-*value
Follow-up time, month	116.56 (2.19)	84.45 (2.03)	** < 0.0001**	112.35 (2.60)	71.62 (4.29)	** < 0.0001**	114.90 (3.77)	71.82 (3.34)	** < 0.0001**
Demographic data									
Age, year	45.79 (0.35)	64.93 (0.37)	** < 0.0001**	41.46 (0.41)	63.22 (1.06)	** < 0.0001**	50.26 (0.64)	67.46 (0.95)	** < 0.0001**
Gender			** < 0.0001**			0.1120			** < 0.0001**
Male	3631 (55.03%)	566 (58.60%)		818 (41.69%)	142 (47.26%)		281 (32.51%)	190 (53.13%)	
Female	3151 (44.97%)	354 (41.40%)		1146 (58.31%)	125 (52.74%)		488 (67.49%)	124 (46.87%)	
Race			0.4047			**0.0454**			**0.0263**
White	2626 (66.91%)	550 (78.07%)		869 (70.47%)	154 (76.47%)		428 (75.88%)	215 (83.01%)	
Black	1462 (11.28%)	200 (11.61%)		497 (12.51%)	67 (13.26%)		145 (9.11%)	56 (9.10%)	
Hispanic	2161 (14.99%)	142 (5.60%)		446 (10.12%)	34 (4.18%)		149 (7.66%)	32 (3.37%)	
Others	533 (6.82%)	28 (4.72%)		152 (6.90%)	12 (6.08%)		47 (7.34%)	11 (4.52%)	
Education level			** < 0.0001**			** < 0.0001**			**0.0001**
Under high school	765 (5.67%)	201 (15.46%)		117 (3.32%)	54 (15.83%)		84 (5.93%)	60 (13.57%)	
High school	2645 (35.33%)	416 (45.36%)		723 (32.17%)	111 (37.20%)		311 (39.76%)	141 (41.19%)	
Above high school	3372 (59.00%)	303 (39.18%)		1124 (64.51%)	102 (46.97%)		374 (54.31%)	113 (45.24%)	
PIR	3.10 (0.06)	2.14 (0.06)	** < 0.0001**	2.97 (0.07)	2.21 (0.13)	** < 0.0001**	2.71 (0.08)	2.24 (0.11)	**0.0008**
Smoking status			** < 0.0001**			** < 0.0001**			** < 0.0001**
Never	3646 (50.75%)	320 (33.09%)		1103 (53.00%)	83 (31.50%)		265 (34.19%)	53 (18.57%)	
Former	1573 (24.79%)	364 (36.71%)		421 (23.67%)	100 (34.44%)		238 (30.19%)	149 (46.52%)	
Now	1563 (24.46%)	236 (30.19%)		440 (23.33%)	84 (34.06%)		266 (35.62%)	112 (34.90%)	
Alcohol consumption			** < 0.0001**			**0.0001**			**0.0169**
Never	4034 (53.95%)	580 (59.76%)		1256 (57.74%)	169 (62.17%)		445 (56.25%)	196 (59.19%)	
Mild	1301 (22.44%)	97 (10.97%)		363 (21.84%)	25 (10.14%)		105 (15.36%)	23 (7.84%)	
Moderate	273 (4.56%)	21 (3.13%)		68 (4.89%)	2 (2.15%)		20 (2.59%)	6 (4.37%)	
Heavy	123 (2.35%)	6 (0.70%)		29 (1.63%)	1 (0.31%)		12 (1.56%)	1 (0.27%)	
Binge drinking daily	1051 (16.70%)	216 (25.45%)		248 (13.89%)	70 (25.23%)		187 (24.24%)	88 (28.33%)	
Body measurement data									
Weight, kg	87.79 (0.44)	83.44 (0.86)	** < 0.0001**	84.30 (0.72)	82.20 (1.46)	0.2069	85.00 (1.00)	81.32 (1.58)	0.0819
Height, cm	169.79 (0.17)	168.30 (0.37)	**0.0001**	168.47 (0.33)	166.51 (0.82)	**0.0212**	166.47 (0.36)	167.82 (1.00)	0.2191
BMI, kg/m^2^	30.41 (0.14)	29.34 (0.30)	**0.0017**	29.70 (0.26)	29.65 (0.67)	**0.9474**	30.65 (0.32)	28.72 (0.48)	**0.0031**
WC, cm	102.57 (0.36)	104.00 (0.67)	**0.0429**	99.62 (0.59)	103.15 (1.15)	**0.0089**	103.15 (0.82)	103.11 (1.36)	0.9850
WWI	11.00 (0.02)	11.46 (0.03)	** < 0.0001**	10.90 (0.03)	11.45 (0.06)	** < 0.0001**	11.25 (0.04)	11.52 (0.07)	**0.0012**
BRI	6.23 (0.05)	6.53 (0.10)	**0.0058**	6.01 (0.09)	6.62 (0.21)	**0.0070**	6.62 (0.11)	6.49 (0.19)	0.5910
ABSI	0.81 (0.00)	0.85 (0.00)	** < 0.0001**	0.81 (0.00)	0.84 (0.00)	** < 0.0001**	0.82 (0.00)	0.86 (0.00)	** < 0.0001**
WHtR	0.61 (0.00)	0.62 (0.00)	**0.0045**	0.59 (0.00)	0.62 (0.01)	**0.0033**	0.62 (0.00)	0.61 (0.01)	0.5494
Comorbidity									
Hypertension	3045 (42.29%)	683 (73.32%)	** < 0.0001**	768 (36.07%)	210 (73.80%)	** < 0.0001**	420 (50.31%)	227 (68.89%)	** < 0.0001**
Diabetes	1226 (14.36%)	344 (32.52%)	** < 0.0001**	325 (12.67%)	92 (31.78%)	** < 0.0001**	189 (19.05%)	111 (30.24%)	**0.0053**
CVD	521 (6.51%)	287 (29.57%)	** < 0.0001**	184 (7.66%)	96 (33.65%)	** < 0.0001**	155 (16.94%)	128 (35.60%)	** < 0.0001**
CKD	317 (4.11%)	267 (26.01%)	** < 0.0001**	73 (3.12%)	74 (22.45%)	** < 0.0001**	54 (6.00%)	92 (25.18%)	** < 0.0001**

Continuous variables are presented as survey-weighted means (SEs), and *p* values were calculated via survey-weighted linear regression. Categorical variables are presented as unweighted counts (weighted percentages), and p values were calculated via the survey-weighted chi-square test. *P* < 0.005 was showed in bold.

*PIR* poverty-income ratio, *BMI* body mass index, *WC* waist circumference, *WWI* weight-adjusted-waist index, *BRI* body roundness index, *ABSI* body shape index, *WHtR* waist‒height ratio, *CVD* cardiovascular disease, *CKD* chronic kidney disease, *OSA* obstructive sleep apnoea, *COPD* chronic obstructive pulmonary disease.

### Relationships between anthropometric indicators and mortality

Table 4 shows the results relating anthropometric indicators to mortality in different population groups. In the OSA group, a one-SD increase in ABSI is linked to an 18% rise in mortality (95% CI 1.09–1.27), while higher BMI and WC are associated with reduced mortality. In the COPD group, increased BMI, WC, and WHtR are linked to lower mortality risks, with BMI showing an HR per SD increment of 0.84 (95% CI 0.72–0.97), WC showing an HR per SD increment of 0.85 (95% CI 0.74–0.97) and WHtR showing an HR per SD increment of 0.86 (95% CI 0.75–0.98). However, in the asthma group, none of the six anthropometric indicators demonstrated significant associations with mortality, suggesting variability in their prognostic value across different respiratory conditions.

**Table 4:** Associations between anthropometric indicators and mortality in the total population, OSA population, and COPD population.

	Adjusted hazard ratio (95% CI)^a^					
	Quartile 1	Quartile 2	Quartile 3	Quartile 4	*P* for trend	Per SD increment
OSA						
BMI	1.00	**0.79 (0.66, 0.94)**	**0.73 (0.61, 0.88)**	**0.74 (0.60, 0.91)**	**0.0034**	**0.89 (0.81, 0.96)**
WC	1.00	**0.76 (0.62, 0.92)**	**0.70 (0.57, 0.85)**	**0.79 (0.65, 0.97)**	0.0626	0.94 (0.87, 1.02)
WWI	1.00	0.81 (0.63, 1.05)	1.01 (0.80, 1.28)	1.15 (0.91, 1.45)	0.0131	1.08 (1.00, 1.18)
BRI	1.00	0.86 (0.71, 1.05)	0.80 (0.65, 0.97)	0.84 (0.68, 1.03)	0.1507	0.96 (0.88, 1.04)
ABSI	1.00	1.02 (0.77, 1.35)	1.09 (0.84, 1.43)	**1.41 (1.08, 1.83)**	**0.0002**	**1.18 (1.09, 1.27)**
WHtR	1.00	0.86 (0.71, 1.05)	0.80 (0.65, 0.97)	0.84 (0.68, 1.03)	0.1192	0.94 (0.87, 1.02)
Asthma						
BMI	1.00	0.92 (0.65, 1.32)	0.89 (0.62, 1.26)	0.79 (0.54, 1.16)	0.2211	0.89 (0.77, 1.04)
WC	1.00	0.75 (0.50, 1.13)	0.86 (0.58, 1.27)	0.75 (0.50, 1.12)	0.3101	0.91 (0.79, 1.05)
WWI	1.00	0.85 (0.50, 1.43)	0.84 (0.51, 1.40)	0.93 (0.55, 1.55)	0.8810	1.01 (0.87, 1.19)
BRI	1.00	0.98 (0.64, 1.48)	1.02 (0.68, 1.52)	0.85 (0.56, 1.30)	0.3712	0.92 (0.79, 1.08)
ABSI	1.00	1.03 (0.60, 1.75)	1.05 (0.63, 1.74)	1.10 (0.66, 1.83)	0.6393	1.06 (0.93, 1.22)
WHtR	1.00	0.98 (0.64, 1.48)	1.02 (0.68, 1.52)	0.85 (0.56, 1.30)	0.3963	0.92 (0.79, 1.07)
COPD						
BMI	1.00	**0.70 (0.51, 0.97)**	0.75 (0.55, 1.02)	**0.66 (0.45, 0.94)**	**0.0352**	**0.84 (0.72, 0.97)**
WC	1.00	**0.71 (0.51, 0.99)**	**0.58 (0.42, 0.81)**	**0.71 (0.50, 1.00)**	**0.0409**	**0.85 (0.74, 0.97)**
WWI	1.00	1.01 (0.71, 1.44)	0.87 (0.61, 1.24)	0.88 (0.61, 1.26)	0.3590	0.93 (0.81, 1.06)
BRI	1.00	0.82 (0.59, 1.13)	0.78 (0.57, 1.08)	**0.70 (0.50, 0.99)**	0.0508	**0.86 (0.75, 0.99)**
ABSI	1.00	0.88 (0.57, 1.37)	1.26 (0.83, 1.89)	1.03 (0.67, 1.57)	0.6717	1.00 (0.87, 1.16)
WHtR	1.00	0.82 (0.59, 1.13)	0.78 (0.57, 1.08)	**0.70 (0.50, 0.99)**	**0.0463**	**0.86 (0.75, 0.98)**

*BMI* body mass index, *WC* waist circumference, *WWI* weight-adjusted-waist index, *BRI* body roundness index, *ABSI* body shape index, *WHtR* waist-to-height ratio, *OSA* obstructive sleep apnoea, *COPD* chronic obstructive pulmonary disease. Statistically significant HRs and* p*-values were showed in bold.

^a^Adjusted for sex, age, race, education level, family poverty-income ratio, diabetes, hypertension, cardiovascular disease, chronic kidney disease, smoking status, and alcohol consumption.

### Prediction performance of the ABSI for 3-year, 5-year and 10-year mortality

**Fig. 2:**
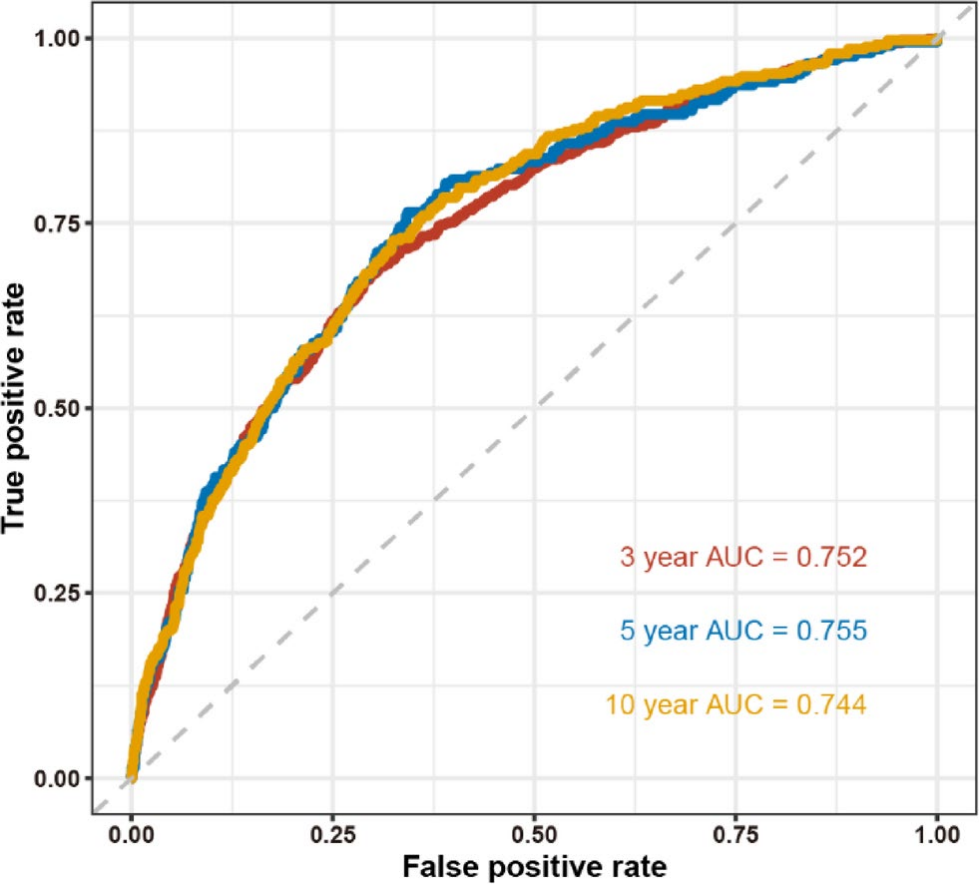
Time-dependent ROC curves of the ABSI for predicting mortality.

**Fig. 3:**
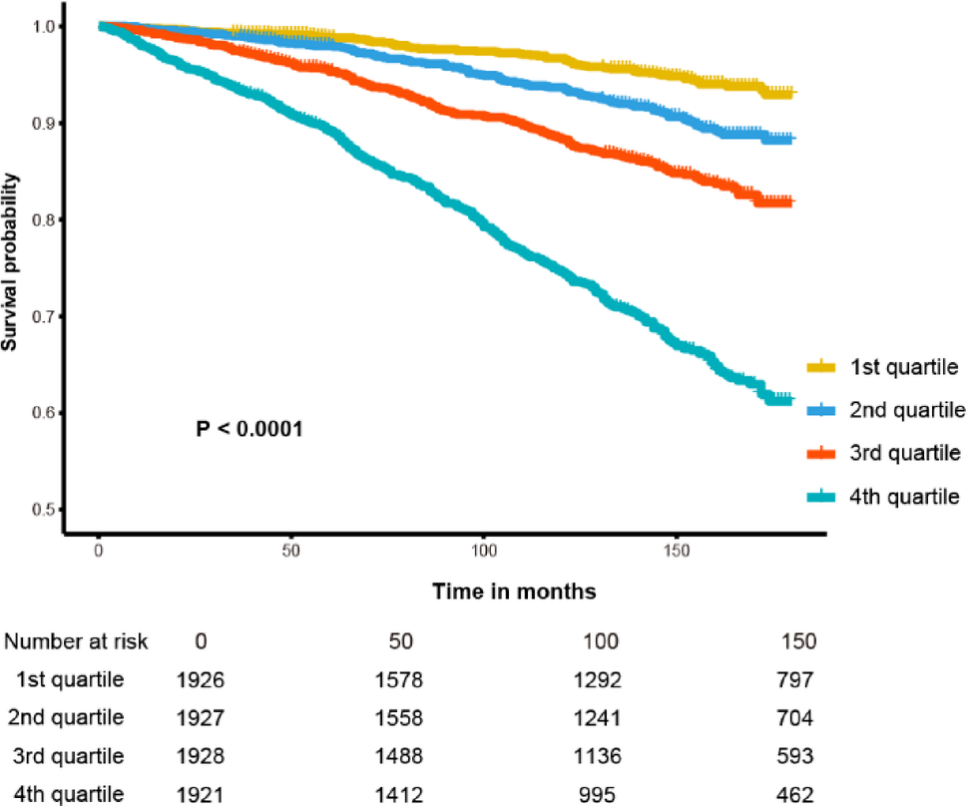
Kaplan–Meier survival analysis across ABSI quartiles.

Figure 2 depicts the time-dependent ROC curves for the prediction of the ABSI for 3-year, 5-year and 10-year mortality in the OSA group. The area under the curve (AUC) for ABSI was 0.752 (95% CI 0.717–0.786) for 3-year, 0.755 (95% CI 0.729–0.782) for 5-year, and 0.744 (95% CI 0.724–0.764) for 10-year mortality. These results underscore the significance of ABSI as a valuable marker for assessing survival prognosis, with superior predictive performance for 5-year mortality. Moreover, Kaplan–Meier survival analyses revealed that individuals in the fourth quartile of ABSI exhibited a notably higher risk of mortality (Fig. 3).

### Stratified analyses

**Fig. 4:**
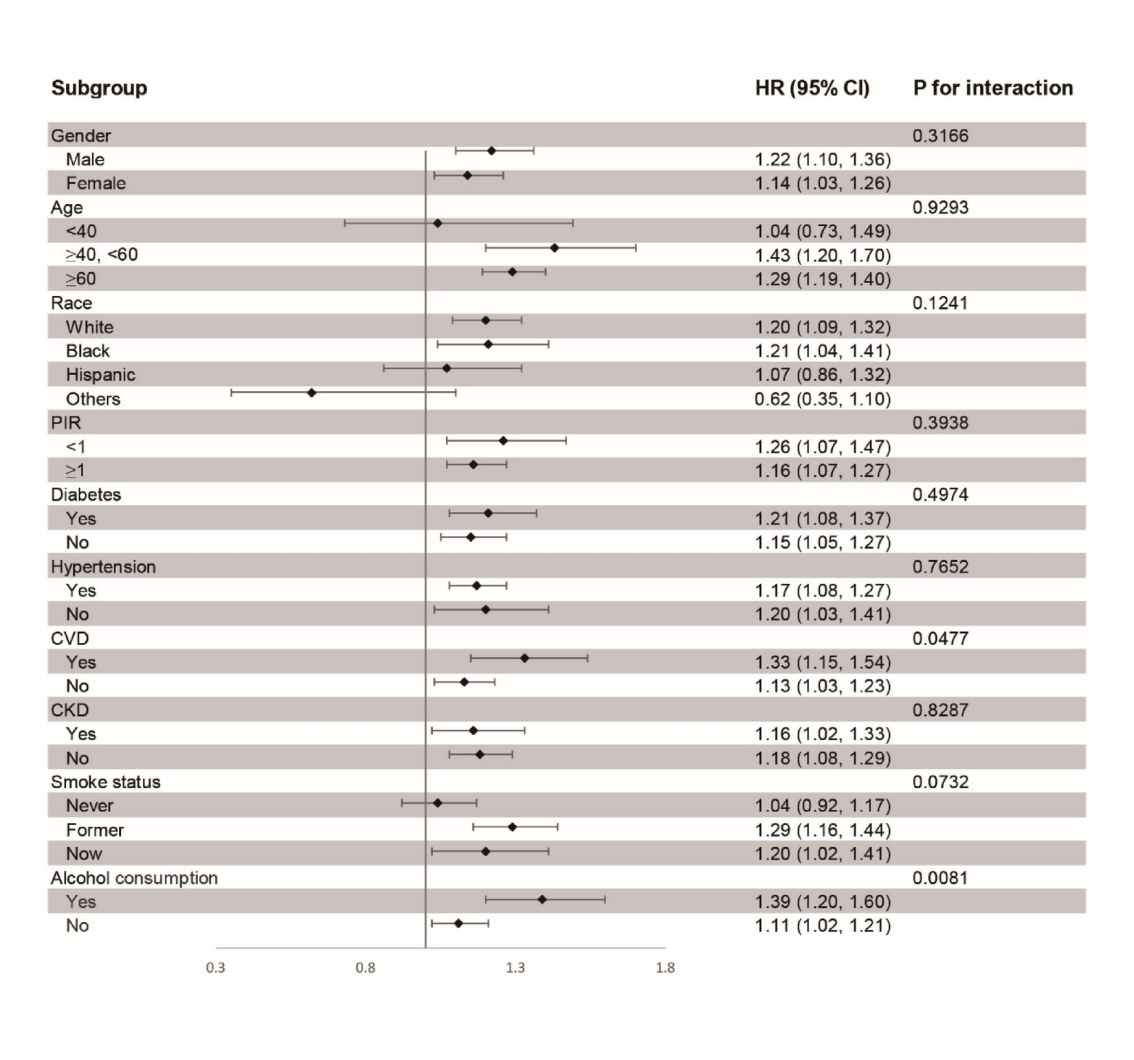
Stratified analysis of the associations between ABSI and mortality among participants with OSA symptoms.

Figure 4 provides insights into the impact of the ABSI on mortality among patients with OSA, across various subgroups. ABSI was found to interact with alcohol consumption (p for interaction = 0.0081) and CVD (p for interaction = 0.0477). A stronger association between ABSI and mortality was observed in alcohol consumers (HR: 1.39, 95% CI 1.20–1.60) compared to non-drinkers (HR: 1.11, 95% CI 1.02–1.21). Similarly, ABSI was more strongly linked to mortality in individuals with CVD (HR: 1.33, 95% CI 1.15–1.54) than those without CVD (HR: 1.13, 95% CI 1.03–1.23). The association between ABSI and mortality was not significant in participants under 40 years old, in those of other races, or in non-smokers.

## Discussion

This study offers a thorough analysis of the predictive performance of various anthropometric indicators in relation to the prevalence of OSA, asthma, COPD, and mortality. All six anthropometric indicators—BMI, WC, WWI, BRI, ABSI, and WHtR—were significantly associated with increased prevalence of OSA and COPD, with particularly robust associations observed for OSA. However, the relationship between asthma and these indicators is more complex: while BMI, WC, BRI, and WHtR are positively correlated with asthma prevalence, ABSI and WWI demonstrate inverse associations. Longitudinal analysis further revealed that traditional anthropometric indicators such as BMI and WC were negatively associated with mortality risks in the OSA and COPD groups, supporting the phenomenon known as the "obesity paradox." However, ABSI emerged as a significant predictor of mortality in OSA, suggesting that the ABSI provides a more nuanced perspective on the impact of central obesity on the risk of death, and may help mitigate the "obesity paradox."

Obesity’s systemic role in chronic diseases is well-documented. Anthropometric indices, particularly BMI and WC, have long been recognized for their connection with the development of respiratory diseases, including OSA, asthma, and COPD. For instance, a community-based cohort study from South Korea revealed that fat mass index and waist-to-hip ratio showed an inverse relationship with lung function in the overall population^22^. Additionally, a meta-analysis concluded that those classified as overweight or obese face a dose-dependent, increased risk of developing asthma^23^. Another cohort study from China revealed that both higher WC and being underweight were linked to a greater risk of developing COPD^24^. This relationship can be attributed, at least in part, to the phenomenon of insulin resistance and the systemic inflammation that occurs in conjunction with obesity, especially abdominal obesity^25^. While our study confirms significant links between newer indices (WWI, BRI, ABSI, WHtR) and OSA/COPD prevalence, the unexpected inverse associations observed between ABSI, WWI, and asthma prevalence suggest a limitation in these indicators’ ability to accurately reflect the impact of obesity on asthma prevalence. This aligns with previous studies that have demonstrated their limited capacity to identify metabolic syndrome^26^ or predicting chronic diseases^27^.

The obesity paradox—wherein obesity appears protective against mortality in chronic diseases—adds further complexity. A meta-analysis of 22 studies comprising 21,150 COPD patients revealed that overweight and obese individuals experienced better survival rates than normal weight people^28^. Another 12-year prospective cohort of Koreans indicated that individuals with lower BMIs had an elevated risk of death from respiratory causes^29^. The survival advantage in obese individuals is complex and disease-specific. One reason for this paradox could be the inherent limitations of the use of BMI as a measurement tool. Our study underscores the limitations of BMI in this context, as its inverse association with mortality contrasts with ABSI’s predictive strength in OSA. Specifically, ABSI offers a more nuanced understanding of how central obesity influences mortality risk in those with OSA. However, in COPD patients, the obesity paradox is even more intricate. This could be attributed to the fact that being underweight, together with loss of muscle mass, represents a principal acute risk element for death in advanced COPD patients^30^. However, recent studies have noted that obese individuals can exhibit low muscle mass, known as sarcopenic obesity, which increases systemic inflammation and worsens quality of life^31^. Therefore, in addition to routine anthropometric measurements, evaluating skeletal muscle function and body composition in COPD patients with an obese phenotype is crucial for understanding the impact of obesity on disease progression^5^.

The clinical relevance of ABSI in OSA warrants particular emphasis. The existing literature presents conflicting perspectives. One single-center study associates ABSI with elevated cardiovascular risk in OSA^32^, while another contradictory study found that BRI is a superior predictor of cardiovascular risk in OSA patients compared to traditional measures, but the ABSI index was not^33^. The novel score, Anthropometric-OSA (A-OSA)—integrating ABSI, lung function, and SpO2—demonstrated enhanced specificity for detecting moderate-severe OSA in surgical patients with obesity compared to the STOP-Bang questionnaire^34^. Critically, our study addresses a pivotal gap: no prior prospective research has directly examined ABSI’s relationship with OSA-related mortality.

These findings advocate for ABSI’s integration into clinical paradigms to refine OSA management. Firstly, ABSI’s prognostic value suggests its utility in identifying high-risk subgroups who may benefit from aggressive interventions, such as continuous positive airway pressure (CPAP) combined with weight-loss programs targeting visceral fat reduction. Secondly, ABSI may aid phenotypic differentiation, distinguishing metabolically unhealthy OSA patients with central adiposity from those with benign fat distribution. Thirdly, in resource-limited settings, ABSI’s simplicity (requiring only waist circumference, weight, and height) makes it a cost-effective tool for longitudinal monitoring. However, its clinical adoption necessitates validation in diverse populations and standardization of measurement protocols to minimize inter-observer variability.

### Strengths and limitations

This study represents the first comparison of the positive associations of BMI, WC, WWI, BRI, and WHtR with OSA, asthma, and COPD. The data were sourced from the NHANES database, a nationwide, population-based survey featuring a large sample size and an extended follow-up period. The application of standardized protocols enhanced the reliability of these findings. Nevertheless, this research presents some inherent limitations. First, the identification of conditions such as OSA, asthma, and COPD relies on self-report questionnaires rather than objective criteria, such as laboratory testing, home sleep apnoea monitoring, or pulmonary function tests, which may have led to inaccuracies in diagnoses and limited our ability to assess disease severity. Second, the cross-sectional study design limits the ability to draw conclusions about causal links between anthropometric measures and disease prevalence. Furthermore, the NHANES dataset provides only baseline anthropometric measurements, meaning dynamic changes in indices over time—which may influence disease progression or mortality—were not captured. These constraints highlight the need for future studies incorporating longitudinal anthropometric tracking and objective diagnostic measures to validate and extend our findings.

## Conclusion

This study demonstrated that novel anthropometric indicators, including WWI, BRI, ABSI, and WHtR, show positive associations with the prevalence of OSA, asthma, and COPD, alongside traditional measures such as BMI and WC. However, WWI and ABSI were more limited in their association with asthma prevalence. Longitudinal analyses revealed that traditional anthropometric indicators such as BMI and WC were negatively associated with mortality risks in the OSA and COPD groups, supporting the phenomenon known as the "obesity paradox." In contrast, ABSI emerged as a significant predictor of mortality in OSA, suggesting that the ABSI provides a more nuanced perspective on the impact of central obesity on the risk of death. However, in COPD patients, routine anthropometric measurements may not fully capture the impact of obesity on the progression of the disease.

## Data Availability

The data examined in this study were sourced from a publicly accessible database, NHANES, available for download from the website: http://www.cdc.gov/nchs/nhanes.htm.

## Abbreviations

NHANES: The US National Health and Nutrition Examination Survey
OSA: Obstructive sleep apnoea
COPD: Chronic obstructive pulmonary disease
BMI: Body mass index
WC: Waist circumference
WWI: Weight-adjusted-waist index
BRI: Body roundness index
ABSI: Body shape index
WHtR: Waist‒height ratio
DXA: Dual-energy X-ray absorptiometry
PIR: Poverty‒income ratio
CVD: Cardiovascular disease
CKD: Chronic kidney disease
SD: Standard deviation
OR: Odds ratio
95% CI: 95% Confidence interval
HR: Hazard ratio
ROC: Receiver operating characteristic
AUC: The area under the curve
